# Dietary Soybean (*Glycine max* (L.) Merr.) Improved the ZP2 Expression in Female *Swiss* Mice

**DOI:** 10.5935/1518-0557.20220020

**Published:** 2023

**Authors:** Ria Margiana, Silvia Werdhy Lestari, Kamila Alawiyah, Pety Narulita, Ahmad Aulia Jusuf, Khoirul Ima

**Affiliations:** 1 Department of Anatomy, Faculty of Medicine, Universitas Indonesia, Indonesia; 2 Master’s Programme in Biomedical Science, Faculty of Medicine, Universitas Indonesia, Indonesia; 3 Research Biobank, Faculty of Medicine, Universitas Indonesia, Indonesia; 4 Cipto Mangunkusumo Hospital, Faculty of Medicine, Universitas Indonesia, Indonesia; 5 Andrology Program, Faculty of Medicine, Universitas Airlangga, Surabaya, Indonesia; 6 Dr. Soetomo General Academic Hospital, Surabaya, Indonesia; 7 Department of Medical Biology, Faculty of Medicine, Universitas Indonesia, Indonesia; 8 Department of Biology, Faculty of Mathematics and Natural Sciences, Sriwijaya University, palembang, Indonesia; 9 Departement of Histology, Faculty of Medicine, Universitas Indonesia

**Keywords:** immunohistochemistry, Mus musculus, ovary follicle cycle, soybean diet, ZP2 expression

## Abstract

**Objective:**

This study aimed to determine the effects of soybean (*Glycine max*) administration on ZP2 expression in female mice.

**Methods:**

This research used *Mus musculus*, six-week-old female SWISS strain mice divided into three groups (group without soybean administration and groups with mixed feed with soybeans and pelleted 50:50 and 25:75). Soybean feed for mice was 360 grams per kilogram of mouse body weight for 2 weeks. The percentage of follicles was measured and analyzed using Hematoxylin-Eosin staining, and the expression of ZP2 was analyzed using immunohistochemistry. We assessed the data using one-way ANOVA and paired t-test using the SPSS 17.

**Results:**

Some of the follicles in the ovaries do not develop until their final stage of follicle maturation. The administration of soybean before and after treatment in all groups was not significantly different, but the numbers of atretic follicles in groups 1 and 2 were significantly different. Soybean administration at a ratio of 50:50 has the effect of increasing the percentage of the ZP2 expression in tertiary follicles (*p*=0.001), whereas soybean administration at a ratio of 25:75 was not able to maintain or increase the formation of ZP2 in tertiary follicles (*p*=0.77).

**Conclusion:**

Soybean administration with a ratio of 50:50 significantly increased the percentage of the ZP2 expression in tertiary follicles.

## INTRODUCTION

The ovaries are the primary reproductive organs that function to produce hormones and female gamete cells. The ovaries have two parts, namely the cortex and the medulla. The oocytes produced in the ovaries are located in the follicles of the ovarian cortex. The development of follicles in the ovaries is called folliculogenesis. This development starts with the primordial follicles until they develop into mature follicles, and the oocytes can be ovulated (Puttabyatappa & Padmanabhan, 2018). Primordial follicles are the earliest follicles and are found after birth. The follicles containing the oocytes are covered by a layer of flat somatic cells. The next stages of the primordial follicles are primary, secondary, and tertiary follicles. Primary follicles form when the oocytes enlarge, and the somatic cell layer changes to a cuboidal shape. This cuboid form will proliferate to form several layers of granulosa cells and the zona pellucida membrane that surrounds the oocytes; these follicles are called the secondary follicles (Cushman *et al*., 2000; Myers *et al*., 2004; Saatcioglu *et al*., 2016). The beginning of tertiary follicles is characterized by the formation of the antrum and the increase in cells around the zona pellucida (ZP) and follicular fluids until the follicles are matured. The increased production of follicular fluid causes the oocytes to be pushed to the edge of the follicles, causing the follicular layer to become thinner, and ovulation occurs (Zuelke & Brackett, 1993). After the ovulation stage, the egg is ready to be fertilized. The egg cell is still covered by the ZP. The fertilization process is strongly influenced by the presence of ZP (Wassarman *et al*., 2001).

ZP is an extracellular matrix crosslinking into a three-dimensional structure that surrounds the oocytes. This is evidenced in the early embryo phase, which consists of four glycoproteins in humans and three glycoproteins in mice, namely ZP1, ZP2, and ZP3. They both have ZP2. ZP plays an important role in the process of oocyte growth and fertilization and the pre-implantation development of the embryo. ZP mediates sperm binding, induces cell acrosome reaction and prevents post-fertilization polyspermy (Da Broi *et al*., 2018). The development of oocytes is influenced by the balance of cellular components of the ovarian follicles. This balance must be achieved for the oocytes to develop properly. Cumulus cells (CCs) and follicular fluids (FFs) are important determinants of oocyte quality. FFs and CCs can support the quality of oocytes and fight the systematic cell destruction condition (Ganguly *et al*., 2010). The functional integrity of FFs and CCs can be determined by a pathological condition that alters the intrafollicular environment (ZPI gene 2020). Damage in oocyte maturation can lead to abnormal egg production. Abnormal eggs are indicated by a non-formed zona pellucida. Moreover, infertility can be associated with ZP2 (Tansey *et al*., 1998; Liu *et al*., 2017).

ZP2 disorder due to genes can be a factor in infertility. In many cases, the protein encoded by ZP2 is responsible for the integrity of the ZP structure and possible mutations in the genes that can cause a defect in oocyte maturation and result in cases of infertility. However, after the fertilization process, normal embryos are indicated by blastocysts and ZP that decompose after ZP is replaced by trophoblast cells. The effects of estrogen after the administration of soybean (G*lycine max*) have been linked to various cardioprotective benefits on adult women in menopause. Previous studies have demonstrated that maternal selenium supplementation with soybeans at various stages of the preconception period can affect the morphology of murine blastocysts and improve implantation status (Da Broi *et al*., 2018). Decreased or damaged follicular reserves and decreased oocyte quality can be associated with oxidative stress and apoptosis.

Isoflavones in soybeans that are an antioxidant and antiapoptotic agents, known to increase ovarian age and oocyte quality. Isoflavones can increase the survival of follicles in female mice (Rai & Jeswar, 2012). Soybean administration can have an effect on menopause women because it has an effect on estrogen. Ovariectomy can be used to determine the effect of dietary soybean estrogens (SBEs) (Tansey *et al*., 1998). Researchers have also investigated the interaction of dietary soybean estrogens with pharmaceutical conjugated estrogens (CEE) in the vagina and uterus. Therefore, the objective of this study was to determine the effect of soybean administration on the expression of ZP2 in female mice. The novelty of this study is that soybean administration can significantly improve oocyte quality in female mice with the expression of ZP2 immunohistochemistry.

## MATERIALS AND METHODS

### Study design

This study used a six-week-old female SWISS strain Mus musculus divided into three different treatment groups. The mice were acclimatized for 7 days before treatment onset. Feeding treatment was carried out for 2 weeks. Group treatment is carried out by giving a combination of pellets and soybeans. The soybeans given are in the form of whole beans. The amount of soybeans given is adjusted to the bodyweight of each mice. The soybean feed was 360 grams per kilogram of mouse bodyweight. Feed was checked and given again every 2 days. The combination of pellet and soybean was 50:50 for group 1 (K1) and a ratio of 25:75 for group 2 (K2). The negative control group was given regular feed with pellets and without soybeans (K3). Total sample sizes were determined using the Federer formula (6/group). The oocyte quality of mice after treatment was analyzed to determine whether giving soybeans could increase or decrease oocyte quality after 2 weeks.

The research was carried out from June 2020 to May 2021 at the Animal House of the Research and Development of the Department of Health, the Histology, Biology, and Anatomy Integration Laboratory (ABH), and at the Department of Anatomy and Histology of FKUI. The acclimatization phase was carried out at the Animal House of the Research and Development Department of Health for 7 days. During the acclimatization period, the mice were fed and given a drink in the form of pellets and water. Mice were then weighed to determine their body weight before the study.

### Oocyte isolation

After 2 weeks of treatment, the mice were weighed again to determine their body weight after the study. The mice were slaughtered in the following way: They were anesthetized with ketamine-xylazine 75-100 mg/kg + 5-10 mg/kg in the IP (intraperitoneal) manner and then euthanized according to the Institutional Animal Care and Use Committee using the method of cervical dislocation, which is done by way of placing the thumb and index finger on either side of the neck at the base of the skull or pressing the trunk to the base of the skull. By holding this part, then the part at the base of the tail was quickly pulled, causing a separation between the skull and neck bones. After that, we made a ventral incision to isolate the ovaries. The ovaries obtained were cleaned of attached fat. The oocytes in the ovaries that had been isolated were then given histological and immunohistochemical staining.

### Histological and immunohistochemical staining analysis

The ovaries that had been immersed in a 10% buffer neutral formalin (BNF) solution for 1-2 days were then made into preparations. The process of making preparations went through the stages of tissue processing, paraffin infiltration and paraffin blocking. The paraffin block containing the ovaries was then sliced with a 5-µm-thick microtome. Tissue preparations were followed by immunohistochemical and histological staining. The staining used was histological staining with Hematoxylin-Eosin (HE). Immunohistochemical staining used the IHK marker, namely rabbit zona pellucida 2 (ZP2) antibody (Genetex, GTXGTX64579). The preparations were photographed with an Optilab camera with a light microscope at 40×, 100×, and 400× magnifications. The resulting photos were processed and analyzed with the ImageJ software.

### Statistical analysis

The data were analyzed using statistical techniques to determine the differences in each experimental group. The data analyzed were the immunohistochemical data for ZP2 and the histological HE. From the HE histological data, we obtained the data on the percentage of follicles for each phase and the area of the ovaries in all groups. Data validation was obtained by calculating the area range of ovaries that could be performed for follicular calculations. This data validation aimed to obtain consistent and not significantly different data so that the data could be compared. The area range of ovaries that was included in the calculation and analysis was 2-3 mm^2^. Data were evaluated using one-way ANOVA and paired t-test on SPSS 17.

### Ethical approval

This study was carried out after its ethics had been reviewed by the Health Research Ethics Committee of the Faculty of Medicine, Universitas Indonesia and obtained a certificate of passing an ethical review, on July 27, 2020, protocol no. 20-07-0777.

## RESULTS

**Figure 1:**
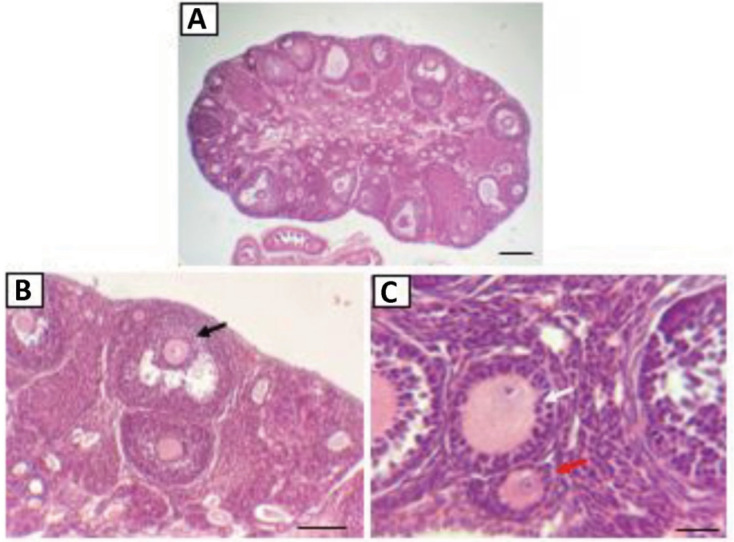
Mice ovaries with Hematoxylin-Eosin (HE) staining. Red arrows are primary follicles; white arrows are secondary follicles; and black arrows are tertiary follicles. Magnification: 40×, scale bar: 100 µm (A); Magnification: 100×, scale bar: 50 µm (B); Magnification: 400×, scale bar: 25 µm (C).

The ovaries play a role in the maturation process of the ovum. In this maturing process, there is a phase that plays a role, namely the folliculogenesis, which begins with the development of primordial follicles until tertiary follicles are mature and ready to ovulate (Figure 1).

**Figure 2:**
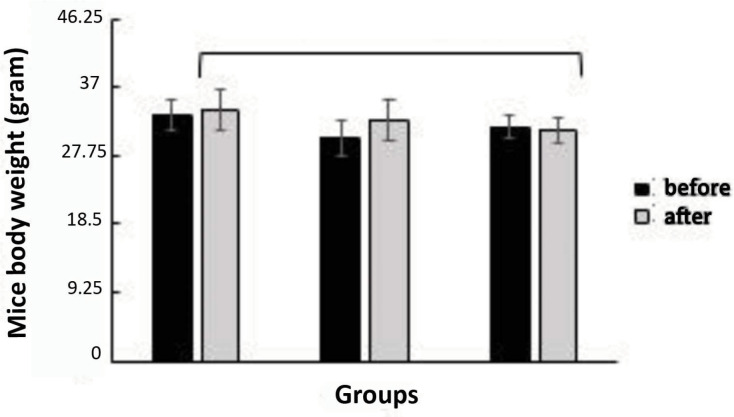
Average body weight of mice in all research groups before and after soybean administration treatment: the group with a 50:50 ratio of soybean to pelleted feed (K1), the group with a 25:75 ratio of soybean to pelleted feed (K2), and the control group without soybean administration (K3). The error bar indicates standard deviation (SD), ts=not significant, one-way ANOVA and paired t-test.

Soybean administration in this study did not change the body weight of mice (Figure 2). The results before and after soybean administration of group 1 (*p*=0.64; paired t-test), 2 (*p*=0.12; paired t-test), and 3 (*p*=0.74; paired t-test) were not significantly different. This indicated that soybean administration does not decrease or increase the body weight of mice.

**Figure 3:**
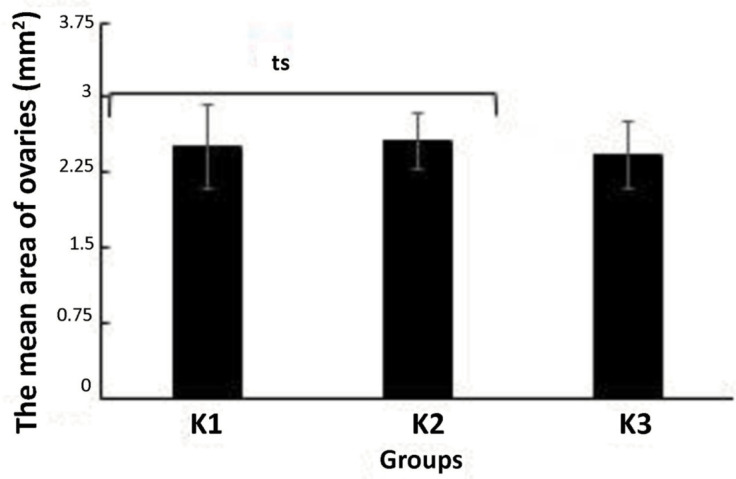
The mean area of the mice ovaries in all study groups after treatment: the group with a 50:50 ratio of soybean to pelleted feed (K1), the group with a 25:75 ratio of soybean to pelleted feed (K2), and the control group without soybean administration (K3). The error bar indicates standard deviation (SD), ts=not significant, one-way ANOVA.

The mean area of ovaries indicated that there were not significantly different results for all the experiment groups (*p*=0.005, one-way ANOVA) (Figure 3).

**Figure 4:**
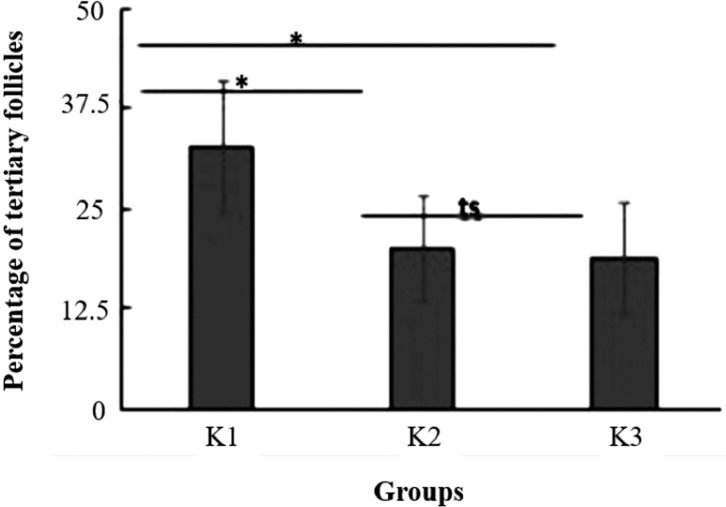
Percentage of tertiary follicles in the ovaries of mice for all study groups: the group with a 50:50 ratio of soybean to pelleted feed (K1), the group with a 25:75 ratio of soybean to pelleted feed (K2), and the control group without soybean administration (K3). The error bar indicates standard deviation (SD). *p<0.05, **p<0.01, ts=not significant, one-way ANOVA.

The percentage of tertiary follicles present in the ovaries in all groups have significantly different values (*p*=0.01; one-way ANOVA) (Figure 4). The percentages of tertiary follicles in group 1 (*p*=0.007; one-way ANOVA) and group 2 (*p*=0.036; one-way ANOVA) are significantly different from that in group 3 (control). However, the percentages of tertiary follicles in groups 1 and 2 do not differ significantly (*p*=0.27; one-way ANOVA).

**Figure 5:**
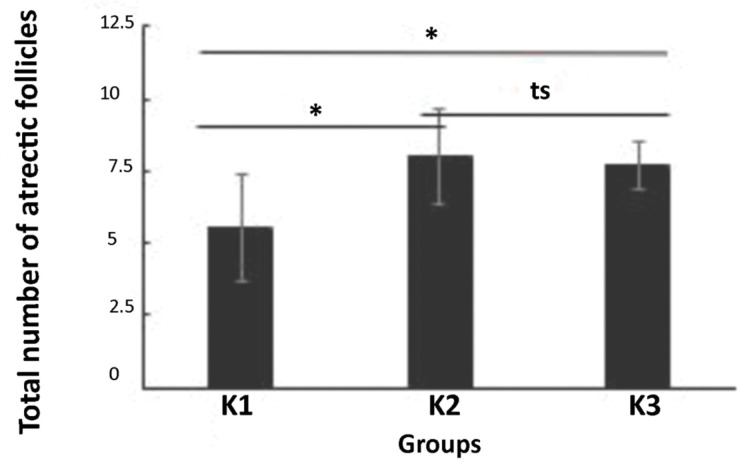
The total number of atretic follicles in the ovaries of mice for all the study groups: the group with a 50:50 ratio of soybean to pelleted feed (K1), the group with a 25:75 ratio of soybean to pelleted feed (K2), and the control group without soybean administration (K3). The error bar indicates standard deviation (SD). *p<0.05, ts=not significant, one-way ANOVA.

Both soybean treatments have the ability to increase the percentage of tertiary follicles. Figure 5 illustrates the number of atretic follicles in all groups. The numbers of atretic follicles in the ovaries of mice for group 1 and group 3 (control) are significantly different (*p*=0.026; one-way ANOVA). This indicated that soybean administration with a ratio of 50:50 can reduce atretic follicles in mice. However, the numbers of atretic follicles in the ovaries of mice for group 2 and group 3 (control) are not significantly different. This indicated that soybean administration at a ratio of 25:75 has not been able to affect atretic follicles in mice.

**Figure 6:**
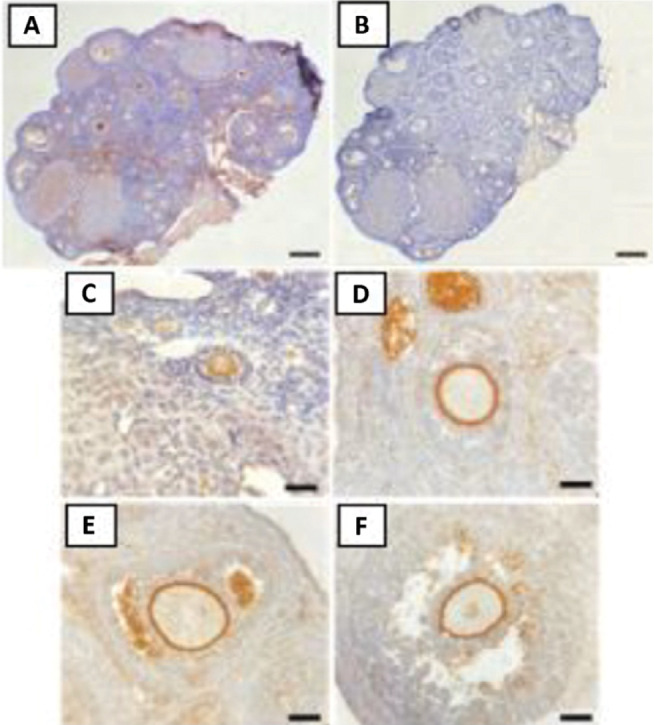
Preparations of mice ovaries with ZP2 marker Immunohistochemical (CPI) staining. Ovaries that were positive (A) and negative (B) for CPI staining with a ZP2 marker, 40× magnification, 100 µm scale bar and primary (C), secondary (D), and tertiary (E and F) follicles that were positive for CPI staining with a ZP2 marker, 400× magnification, 25 µm scale bar.

Figure 6 indicated that ZP perfectly surrounds the oocytes. Along with the development of the follicles in the tertiary/mature phase, ZP will get thicker and will be followed by an increase follicle size. The immunohistochemistry staining results on ZP2 is illustrated in Figure 6.

**Figure 7:**
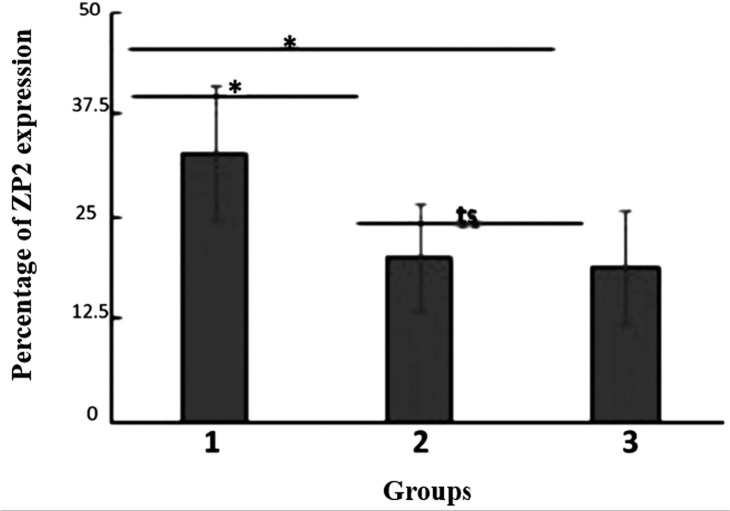
The percentage of ZP2 expression in tertiary follicles of mice ovaries for all the study groups. The group with a 50:50 ratio of soybean to pelleted feed (K1), the group with a 25:75 ratio of soybean to pelleted feed (K2), and the control group without soybean administration (K3). The error bar indicates standard deviation (SD). * *p*<0.05, **p<0.01, ts=not significant, one-way ANOVA.

The results indicated that there was a significant difference between group 1 and group 3 (*p*=0.001; one-way ANOVA), whereas in groups 2 and 3, there was no significant change (*p*=0.77; one-way ANOVA). This indicated that soybean administration at a ratio of 50:50 has the effect of increasing the percentage of ZP2 expression in tertiary follicles. There was a significant difference between groups 1 and 2 (*p*=0.01; one-way ANOVA) (Figure 7).

## DISCUSSION

Some of the follicles in the ovaries will not develop until the final stage of follicle maturation. Some of the follicles will experience atresia. Atresia is a process where follicles do not develop into maturation. Atretic follicles can be found at any stage of follicular development. The follicles counted and analyzed in this study were primary, secondary, tertiary, and atretic follicles. Each phase has morphological differences indicated by the histology results with HE (Figure 1). Each phase difference in the ovaries indicates a development of the follicular phase in the ovaries. Based on Figure 1, each follicular phase is found in a specific area of the ovaries (Figure 1A). Primary follicles are generally found in the ovarian cortex, whereas secondary and tertiary follicles are found throughout the ovaries. In this study, the specific characteristic of the primary follicles used is the presence of a layer of cuboidal granulosa cells and flat cells in the outer area surrounding the oocytes and the initial formation of the zona pellucida. The secondary follicles are characterized by more than one layer of cuboidal granulosa cells surrounding the oocytes (Figure 1C). Tertiary follicles are also characterized by the presence of more than one layer of cuboidal granulosa cells. However, the difference is that in the tertiary follicles, the antrum begins to form (Figure 1B).

The antrum is a cavity within the follicles. This cavity is formed from the secretion of follicular fluids by granulosa cells. The tertiary follicles calculated in the study are from the formation of the antrum to the Graafian follicles in the maturation of the oocytes. Follicles that fail to develop until the oocytes mature are categorized as atretic follicles. The grouping and calculation of each follicular phase refers to Myers *et al*. (2004) and Scudamore (2014). After follicle maturation takes place, the next stage occurs, namely ovulation. Ovulation occurs when an ovum leaves the ovaries. The granulosa cells in the follicles that are left behind by the ovum will undergo proliferation at a magnification that contains many luteins, capillaries and connective tissues, until the corpus luteum is formed. The corpus luteum will secrete the hormone progesterone in large quantities. Progesterone prepares the uterus for implantation, so the corpus luteum will be maintained if there is fertilization until the placenta is fully formed. Many factors influence the level of ovum maturation and fertility of the ovaries. One of them is body weight (Figure 2).

Weight gain can affect fertility rates and the ovulation process. Being over- and under-weight has been linked to ovulatory infertility. This sub-optimal body weight will result in subfertility, polycystic ovary syndrome, and irregular menstrual cycles (Alchami *et al*., 2015; Vitek *et al*., 2020). From the results of the study, soybean administration did not have an effect on body weight changes in the mice (Figure 2). The results before and after soybean administration in groups 1 (*p*=0.64; paired t-test), 2 (*p*=0.12; paired t-test), and 3 (*p*=0.74; paired t-test) are not significantly different. This indicated that soybean administration does not decrease or increase the mice body weight.

Calculation and analysis in this study should be followed by data validation so that there is bias in the study results. The validation of the data used in the calculation of the results of this study was carried out by calculating the total area of the ovaries of the mice in all the study groups. The calculation of the area of the ovaries as a whole used the Imagej application. This calculation was aimed at determining the ovarian inclusion criteria that was then used in calculating the follicular phase in the ovaries. The calculation and analysis did not indicate significantly different results for the whole group (*p*=0.005, one-way ANOVA) (Figure 3). This indicated that the calculation and data analysis in the study were consistent and comparable. The area of the ovaries, which was used as a calculation (inclusion) criterion, was ±2 mm^2^.

The results of the study on the percentage of tertiary follicles present in the ovaries in all groups have significantly different values (*p*=0.01; one-way ANOVA) (Figure 4). The percentages of tertiary follicles in group 1 (*p*=0.007; one-way ANOVA) and group 2 (*p*=0.036; one-way ANOVA) are significantly different from that in group 3 (control). This indicated that soybean administration can increase the percentage of tertiary follicle maturation. However, the percentages of tertiary follicles in groups 1 and 2 do not differ significantly (*p*=0.27; one-way ANOVA). This indicated that the ratio of the feed to soybeans does not affect the percentage of tertiary follicles. Both soybean ratio comparisons have the ability to increase the percentage of tertiary follicles. Figure 5 illustrates the number of atretic follicles in all groups. The numbers of atretic follicles in the ovaries of mice for group 1 and group 3 (control) are significantly different (*p*=0.026; one-way ANOVA). This indicated that soybean administration at a ratio of 50:50 can reduce atretic follicles in mice. However, the numbers of atretic follicles in the ovaries of mice for group 2 and group 3 (control) are not significantly different. This indicated that soybean administration at a ratio of 25:75 has not been able to affect atretic follicle outcomes in mice. The numbers of atretic follicles in the ovaries of mice for group 1 and group 2 are significantly different. This indicated that soybean administration at a ratio of 50:50 has a larger effect than that of soybean administration at a ratio of 25:75 on the reduction of atretic follicles in mice.

The formation of the follicles from the beginning to maturity is followed by the death of the follicles or atretic follicles. Atretic follicles aim to balance the number of follicles in the ovaries (Prakash *et al*., 2007; Strauss & Williams, 2009). However, too many follicular deaths and damages should be inhibited. The development of the follicles is influenced by many factors inside and outside the body. One of the body-related factors is the hormone (De los Reyes *et al*., 2006). The external factor is food intake. The administration of soybeans is an external factor for fulfilling nutrition. Soybeans have a fairly high antioxidant content. Antioxidant agents are needed by the body to protect against free radicals. These free radicals can damage and inhibit follicle formation and cause damage to granulosa cells. The process of inhibition and breakdown of granulosa cells causes a decrease in atretic follicles. Soybean is a widely consumed food that acts as an antioxidant due to its high isoflavone content (Atho’illah *et al*., 2019). Isoflavones work similarly to estrogens, needed in optimal amount, not too little, not too much (Lima *et al*., 2014; Uyar *et al.,* 2014). Therefore, the amount, level, and dose administration have an effect on decreasing and increasing the number of follicles l.

The results indicated that there was a significant difference between group 1 and group 3 (*p*=0.001; one-way ANOVA), whereas between groups 2 and 3, there was no significant difference (*p*=0.77; one-way ANOVA). This indicated that soybean administration at a ratio of 50:50 increases the percentage of ZP2 expression in tertiary follicles. There was a significant difference between groups 1 and 2 (*p*=0.01; one-way ANOVA) (Figure 7). This indicated that soybean administration at a ratio of 25:75 has not been able to maintain or increase ZP2 formation in tertiary follicles. ZP formation is influenced by many factors, including nutrition. In this study, soybeans were used in mice feed. The results indicated a ZP2 increase in tertiary follicles after soybean administration. Soybeans contain isoflavonoids that are good for oocyte development and ZP formation (Liu *et al*., 2017). Good ZP formation will affect oocyte maturation. ZP will survive and be used starting from the initial formation of follicles to the implantation stage in the uterine wall. At the initial formation and maturation of follicles, ZP will be produced by oocytes (Schroeder *et al*., 1990; Wang *et al*., 2019). This will indicate that these oocytes are healthy and may become mature. During the fertilization phase, ZP will be used as a barrier for the sperms that are going to fertilize the ovum. The role of ZP2 is to serve as a secondary sperm receptor, especially after the induction of the initial sperm acrosome reaction. After fertilization, ZP2 functions in the initial block of polyspermy after initial contact with ZP3. ZP2 will maintain contact between the ovum and sperms. Knockout ZP2 causes infertility (Rankin *et al*., 2001; Liu *et al*., 2017; Dai *et al*., 2019). Therefore, ZP2 has very important roles to play. ZP2 is an amino-acid glycoprotein 745 (Litscher & Wassarman, 2020). The roles and functions of ZP are not only in the initial process of fertilization but also during folliculogenesis and embryo development. ZP will protect the oocytes and early embryos formed from the outside environment. This protection prevents infections caused by microbes, bacteria and viruses (Wrathall, 1995). ZP2 formation starts at the primary follicle formation. ZP is formed by the oocytes themselves (Dunbar *et al*., 1994). The location of ZP is surrounding the oocytes and it is surrounded by granulosa cells. ZP that perfectly surrounds oocytes is one indication of healthy follicles. Along with the development of the follicles in the tertiary/mature phase, ZP will get thicker and will be followed by an increase in follicle size. Therefore, oocyte quality is associated with zona pellucida. The representation of the staining results on ZP2 is provided in Figure 6.

## CONCLUSION

Soybean administration at a ratio of 50:50 significantly increased the percentage of the ZP2 expression in tertiary follicles.
